# The Molecular and Pharmacological Mechanisms of HIV-Related Neuropathic Pain

**DOI:** 10.2174/1570159X11311050005

**Published:** 2013-09

**Authors:** Shuanglin Hao

**Affiliations:** Department of Anesthesiology, University of Miami Miller School of Medicine, Miami, FL33136

**Keywords:** HIV, neuropathic pain, gp120, NRTI, spinal cord, DRG.

## Abstract

Infection of the nervous system with the human immunodeficiency virus (HIV-1) can lead to cognitive, motor and sensory disorders. HIV-related sensory neuropathy (HIV-SN) mainly contains the HIV infection-related distal sensory polyneuropathy (DSP) and antiretroviral toxic neuropathies (ATN). The main pathological features that characterize DSP and ATN include retrograde (“dying back”) axonal degeneration of long axons in distal regions of legs or arms, loss of unmyelinated fibers, and variable degree of macrophage infiltration in peripheral nerves and dorsal root ganglia (DRG). One of the most common complaints of HIV-DSP is pain. Unfortunately, many conventional agents utilized as pharmacologic therapy for neuropathic pain are not effective for providing satisfactory analgesia in painful HIV-related distal sensory polyneuropathy, because the molecular mechanisms of the painful HIV-SDP are not clear in detail. The HIV envelope glycoprotein, gp120, appears to contribute to this painful neuropathy. Recently, preclinical studies have shown that glia activation in the spinal cord and DRG has become an attractive target for attenuating chronic pain. Cytokines/chemokines have been implicated in a variety of painful neurological diseases and in animal models of HIV-related neuropathic pain. Mitochondria injured by ATN and/or gp120 may be also involved in the development of HIV-neuropathic pain. This review discusses the neurochemical and pharmacological mechanisms of HIV-related neuropathic pain based on the recent advance in the preclinical studies, providing insights into novel pharmacological targets for future therapy.

## 1. INTRODUCTION

Typically transient pain (acute pain) that occurs in response to noxious stimuli (nociceptive pain) is early-warning protective, and is mediated by specialized high-threshold primary sensory neurons. Chronic pain, is associated either with tissue damage and inflammation (inflammatory pain) or with lesions to the nervous system (neuropathic pain), characterized by persistent pain. These include, pain experienced in the absence of any obvious peripheral stimulus (spontaneous pain), an increased responsiveness to noxious stimuli (hyperalgesia), and/or pain in response to normally innocuous stimuli (allodynia) [1]. A classification relating to the type of neuropathic disorders, includes mechanical nerve injury (e.g. carpal tunnel syndrome, vertebral disk herniation); metabolic disease (e.g. diabetic polyneuropathy), neurotrophic viral disease (e.g. herpes zoster, human immunodeficiency virus (HIV)), neurotoxicity (e.g. chemotherapy of cancer or tuberculosis), inflammatory and/or immunologic mechanisms (e.g. multiple sclerosis), nervous system focal ischemia (e.g. thalamic syndrome) and multiple neurotransmitter system dysfunctions (e.g. complex regional pain syndrome), etc. [2].

HIV-related neuropathic pain is a debilitating chronic condition that is severe and unrelenting. Despite decades of extensive research, the neuropathological mechanisms remain unknown in detail, hindering our ability to develop effective treatments. This review focuses on current researches on the pathophysiological mechanisms of HIV-neuropathic pain.

## 2. PERIPHERAL AND CENTRAL SENSITIZATION OF NEUROPATHIC PAIN

In the last 20 years, significant basic research progress has been made in developing and characterizing *in vivo* experimental models of chronic pain. Two main mechanisms of neuropathic pain, peripheral and central becomes sensitizations, not mutually exclusive, are proposed.

### 2.1. Peripheral Sensitization

Primary afferent fibers transmit noxious stimuli from the periphery to the central nervous system. In addition, primary afferent fibers have a unique morphology, called pseudo-unipolar, wherein both central and peripheral terminals emanate from a common axonal stalk [3]. Therefore, the majority of proteins synthesized by the dorsal root ganglions (DRG) is distributed to both central and peripheral terminals [3]. Tissue damage or inflammation is often accompanied by the accumulation of endogenous factors released from activated nociceptors or non-neural cells that reside within or infiltrate into the injured area [3-5]. Collectively, these factors represent a wide array of signaling molecules, including neurotransmitters, peptides (substance P, bradykinin), eicosanoids and related lipids (prostaglandins, etc.), neurotrophins, proinflammatory cytokines (interleukin-1β (IL-1β) and IL-6, and tumor necrosis factor α (TNF-α)), and chemokines, as well as extracellular proteases and protons, referred to as the ‘inflammatory soup’ [4]. These factors act directly on the nociceptors by binding to one or more cell surface receptors, including G protein-coupled receptors (GPCR), Transient receptor protein (TRP) channels, acid-sensitive ion channels (ASIC), two-pore potassium channels (K2P), and receptor tyrosine kinases (RTK), as depicted on the peripheral nociceptor terminals [3]. Nerve growth factors (NGF) or proinflammatory cytokines-induced activation of mitogen-activated protein kinases (MAPK) in primary sensory neurons exacerbates hyperalgesia [6, 7]. Transient receptor protein vanilloid 1 (TRPV1) is a key component of the mechanism through which inflammation produces thermal hyperalgesia modulated by components of the inflammatory soup [8]. Some of these inflammatory agents (for example, extracellular protons and lipids) function as direct positive allosteric modulators of the channel, whereas others (bradykinin, ATP, and NGF) bind to their own receptors on primary afferents and modulate TRPV1 through activation of downstream intracellular signaling pathways. These factors result in functional potentiation of target proteins at the peripheral nociceptor terminal, leading to a rapid change in cellular and behavioral sensitivity [9]. This increases the sensitivity and excitability of the nociceptor terminal–a phenomenon known as peripheral sensitization [1, 4], which produces increases in pain sensitivity that is restricted to the site of inflammation. 

### 2.2. Central Sensitization

Central sensitization refers to the process through which a state of hyperexcitability is established in the central nervous system, leading to enhanced processing of nociceptive (painful) messages [10]. Although many mechanisms have been implicated in the central sensitization, there are at least three main aspects involved in the sensitization: glutamatergic neurotransmission/N-Methyl-D-aspartate (NMDA) receptor-mediated hypersensitivity, loss of tonic inhibitory controls (disinhibition), and glial-neuronal interactions [3]. 

In the spinal dorsal horn, primary afferent C/Aδ fibers release peptide (e.g., substance P/ calcitonin-gene related peptide (CGRP), etc.) and excitatory amino acid (glutamate) products. Acute pain is signaled by the release of glutamate from the central terminals of primary afferent nociceptors, generating excitatory postsynaptic current in second order dorsal horn neurons, which occurs primarily through activation of postsynaptic glutamatergic α-amino-3-hydroxy-5-methylisoxazole-4-propionic acid (AMPA) and kainate subtypes of ionotropic glutamate receptors [3]. Electro-physiologically, glutamatergic AMPA receptor antagonists diminish small afferent-evoked excitation; for glutamate, direct monosynaptic excitation is mediated by non-NMDA receptors (i.e., AMPA receptor) [11]. The NMDA subtype of glutamate channel is silent in normal condition. NMDA antagonists do not appear to reduce monosynaptically mediated afferent-evoked excitation and thus are not believed to be immediately postsynaptic to the primary afferent terminal, though some binding may be on the C-fiber terminal itself. However, in the setting of injury, increased release of neurotransmitters from primary afferent nociceptors sufficiently depolarizes postsynaptic neurons to activate NMDA receptors in second-order neurons [11]. The consequent increase in calcium influx can strengthen synaptic connections between nociceptors and dorsal horn pain transmission neurons, which in turn exacerbate responses to noxious stimuli [3]. A host of downstream signaling pathways and second messenger systems, notably kinases (such as MAPK, protein kinase A, protein kinase C, Phosphoinositide 3-kinase), is involved in the excitability of these neurons [12, 13].

The main type of inhibitory synaptic transmission in the dorsal horn is mediated by *γ*-aminobutyric acid (GABA) and glycine receptors, which are ligand-gated Cl^-^ channels. Under normal conditions, the inhibitory control on the pain system is very powerful, serving as a ‘gate control’ to maintain the balance between excitatory and inhibitory synaptic inputs [14, 15]. But, in the setting of injury, this inhibition may be lost, resulting in disturbance of the balance leading to abnormal pain sensitivity [16, 17]. Meanwhile, the disinhibition can enable non-nociceptive Aβ afferents to engage the pain transmission circuitry such that normally innocuous stimuli are now perceived as painful [3, 15, 18-21].

Research on glial cells has come of age. The outdated concept that glia were simply the glue that holds the nerve cells together but otherwise have no active role in the brain, has been laid to rest for good [22]. The role of glia in, among other examples, synapse formation, synapse maturation and plasticity, and the rapid conduction of action potentials, as well as their immunological functions in the nervous system, has by now been unequivocally established [22]. Peripheral nerve injury/inflammation promotes release of neurotransmitters and neuropeptides that stimulate glial cells. Microglial activation occurs within minutes but can be long-lasting [23]. Activation of at least 5 major paths including fractalkine, interferon-γ, monocyte chemoattractant protein-1, toll-like receptor 4 (TLR4), and P2X on microglia, is involved in certain neuropathic nociceptive states [24]. Glia release brain-derived neurotrophic factor (BDNF), and a host of cytokines, such as TNFα, IL-1β and IL-6, and other factors (e.g. chemokines), which through their receptors expressed by neurons in the spinal dorsal horn, promotes increased excitability and enhanced pain in response to both noxious (hyperalgesia) and innocuous stimulation (allodynia) [3, 13, 25, 26]. Astrocytes have been recently identified as important components of the tripartite synaptic complex [27]. There is growing evidence that astrocytes regulate synaptic functions of neurons, in part, through the release of gliotransmitters [28, 29]. 

## 3. HIV-ASSOCIATED SENSORY NEUROPATHY AND PAIN

Since the first report of HIV/AIDS was published in the United States in 1981, the Centers for Disease Control and Prevention estimates that more than 1.8 million people in the U.S. have been infected with HIV, and more than 1.1 million estimated to be living with the disease today. While the number of new HIV infections was down from its peak in the 1980s, there have been approximately 50,000 new cases occurring every year [30]. Patients with HIV infection have numerous complications including neurological disorders. HIV-associated sensory neuropathies (HIV-SN) are the most common form of peripheral neuropathy, affecting about 30% of adults and children with AIDS [31, 32]. Sensory neuropathies that are a sequel of HIV infection, are known as distal sensory polyneuropathy (DSP), whereas the sensory neuropathies that result from antiretroviral therapy (ART), are known as antiretroviral drug-induced toxic neuropathies (ATN) [33]. 

The most common complaint of HIV-DSP is pain on the soles; the pain is typically bilateral, of gradual onset, and described as ‘aching’, ‘painful numbness’, or ‘burning’[34]. Patients often have hyperalgesia and allodynia in a stocking and/or glove distribution. The feet are tender to touch, wearing shoes is painful, and the gait becomes ‘antalgic’. In a typical length-dependent fashion, the dysesthesias ascend proximally up the lower extremities over months, and may begin to involve the fingertips at around the same time as they reach the mid-leg level [33, 35]. It is usually most severe on the soles of the feet, and is typically worse at night. 

Pathologically, the most common histological feature of both DSP and ATN is characterized by loss of DRG sensory neurons, Wallerian degeneration of the long axons in distal regions, DRG infiltration by HIV-infected macrophages, and a 'dying back' sensory neuropathy [36-40]. Early on, small, unmyelinated sensory fibers are lost, with eventual destruction of the large myelinated fibers as the disease progresses in the patients with HIV. In the periphery and the DRG, there is infiltration of macrophages and other inflammatory cytokines [41]. Clinically, these two forms (HIV-DSP and ATN) of HIV sensory neuropathies are difficult to distinguish.

### 3.1. Neurochemical Mechanisms of HIV-DSP Neuropathic Pain

HIV-related neuropathic pain is a debilitating chronic condition that is severe and unrelenting. Despite decades of extensive research, the neuropathological mechanisms responsible for the development of this devastating condition remain largely unknown. Aberrant glia-neuron signaling is revealed in the pathogenesis of neuro-AIDS in HIV-1 infected individuals. Infected microglia release products that can be defined as “virotoxins” [42] consisting of toxic viral proteins, and inflammatory “cellular toxins” [43]. While astrocytes contributes less than microglia sources of virotoxins [44], astrocytes can be activated by cellular toxins from microglia. Viral protein gp120 is HIV viral exterior envelope glycoprotein cleaved from gp160. The entry of HIV into cells requires sequential interaction of gp120 with CD4 glycoprotein and chemokine receptors (CXCR4 and/or CCR5 as co-receptors of gp120) on the cell surface [45-48], to induce neurological dysfunction [49-52]. HIV gp120 exerts both direct and indirect neurotoxic effects in the nervous system through the release of proinflammatory factors [53]. A direct role of gp120 in the genesis of the neuropathic pain of DSP has been suggested. In 1996, Eron *et al*., reported that HIV-infected patients experienced pain at the injection site while they investigated whether immunization of patients who had symptomless HIV-1 infection with an envelope subcomponent vaccine (MNrgp120) to augment immune response, could slow the progression of HIV-1 disease [54]. Intrathecal administration of recombinant gp120 induces robust thermal hyperalgesia and mechanical allodynia [55]. Oh and colleagues have demonstrated that cultured rat DRG neurons express a wide variety of chemokine receptors including C-X-C chemokine receptor 4 (CXCR4), and that gp120 injected into the rat paw induces allodynia, providing evidence that chemokines and gp120 produce painful effects *via *direct actions on chemokine receptors expressed by nociceptive neurons [56]. Recombinant gp120 transiently delivered epineurally *via *oxidized cellulose wrapped around the rat sciatic nerve [57], induces mechanical allodynia and thermal hyperalgesia. Since then, these models have been using to investigate the molecular mechanisms of gp120-induced pain and the efficacy of potential therapeutic interventions. 

#### 3.1.1. Proinflammatory Cytokines 

It is known that proinflammatory cytokines (e.g. TNFα, IL1-β, and IL-6, etc.) play an important role in the development and maintenance of inflammatory and neuropathic pain in preclinical studies [58-62]. Lymphocyte and macrophage infiltration is seen within the DRG of AIDS patients, with concomitant presence of pro-inflammatory cytokines including TNFα, interferon-γ (IFN-γ), IL-1 and IL-6 [38, 41, 63-66]. The increased levels of IL-1β and TNFα in human CSF [67-71] and brain tissue [71-74] have been reported in patients with HIV-1. HIV-1-infected patients have significantly higher plasma levels of TNFα and interleukin-6 [75]. An elevated baseline TNFα level among HIV-1 positive individuals may lead to additional neurodegeneration [76]. It is believed that the nerve injury initiates a cascade of events that lead to the development of chronic pain in patients with HIV [77]. Thus, the early presence of cytokines may be involved in the induction and/or progression of DSP neuropathic pain.

The HIV virus infection is able to increase the production and utilization of several cytokines, such as TNFα, IL-1, or IL-6 [78]. Kitano *et al* reported the HIV viral production is suppressed in the presence of anti-TNF antibodies in *in vitro* study [79]. The production of TNFα, a pro-apoptotic cytokine, uses mitochondria as targets [80]. Buch and colleagues have reported that the interplay of TNFα and HIV-1 leads to enhanced expression of the toxic chemokine [81]. Maggirwar and colleagues have showed that HIV protein influences neuronal survival by increasing in TNFα production [82]. Studies show a difference in neuropathogenic manifestations of HIV-1 neuroAIDS between HIV-1 subtypes, clade B- and clade C---infected subjects with clade B being more neuro-pathogenic than clade C; monocytes treated with clade B protein show a significant upregulation of proinflammatory cytokines, TNFα and IL-6, as compared to clade C protein-treated cultures [83]. 

In *in vivo* studies, HIV-1 transgenic rats overexpressing gp120, induces reactive gliosis in brain [84]. Intrathecal administration of soluble gp120 induces neuropathic pain and proinflammatory cytokine release in the spinal cord [85]. The gp120-exposed sciatic nerve exhibits pathology, notably axonal swelling and increased TNFα within the nerve trunk [57]. And, intense astrocytic and microglial activation is observed in the spinal cord, and this gliosis persisted for at least 30 days following epineural gp120, in parallel with neuropathic pain behaviors [57]. We and others have reported that application of recombinant gp120 to the sciatic nerve increases TNFα in the DRG and spinal cord [57, 86]. Peripheral gp120 application into the rat sciatic nerve upregulates the expression of spinal TNFα in the mRNA and the protein levels in the microglia and astrocytes at 2 weeks after gp120, and increases TNFα in the L4/5 DRG [86]. Furthermore, intrathecal TNFα siRNA or soluble TNF receptor reduces gp120 application-induced mechanical allodynia, indicating that TNFα in the spinal cord and the DRG are involved in neuropathic pain induced by HIV gp120 [86]. 

HIV neurotoxicity is mediated through the pro-inflammatory cytokine IL-1β. IL-1β produced by neurons in response to gp120 ligation of CXCR4, acted in an autocrine fashion to sensitize neurons to excitotoxicity [87-89]. The actions of IL-6 in gp120-evoked neuropathic pain states appear to be pro-nociceptive in nature [90]. Watkins and colleagues have demonstrated that blockade of IL-6 abolishes gp120-induced mechanical allodynia and inhibits gp120-mediated increases in TNF, IL-1, and IL-6 mRNA in the dorsal spinal cord, as well as TNFα and IL-1β protein release into the surrounding cerebrospinal fluid [90].

TNFα and IL-1β may act at least partly *via *MAPK cascades in glia [91-93]. Systemic administration of CNI-1493 (a p38MAPK inhibitor), blocks centrally mediated thermal hyperalgesia and mechanical allodynia induced by intrathecal gp120 (most likely *via *interfering with proinflammatory cytokine signal transduction) [94]. 

#### 3.1.2. Chemokines 

Chemokines are chemotactic cytokines that were originally discovered as promoters of leukocyte proliferation and mobility. Considering the widespread expression of CXCR4 and other chemokine receptors in the nervous system, CXCR4 is an important factor in the neuro-pathogenesis of HIV/AIDS [95]. In *in vitro* studies, binding of gp120 to CXCR4 receptors expressed by DRG satellite glial cells, upregulates the release of the Regulated on Activation, Normal T cell Expressed and Secreted (RANTES) chemokine (also known CCL5), which then activates CCR5 receptors expressed by DRG neurons to produce TNFα and subsequent TNFR1-mediated neurotoxicity in an autocrine fashion [37]. On the other hand, gp120 binds to and activates CXCR4 expressed by the DRG neurons in CD-4-independent manner [56, 96] to produce Ca2^+^-dependent upregulation of chemokine (C-C motif) receptor 2 (CCR2) expression by these neurons, suggesting direct neurotoxic effects of gp120 on neurons [97]. In *in vivo *studies, unilateral administration of gp120 into sciatic nerve, induces profound tactile hypernociception; monocyte-chemoattracting protein 1 (MCP1) and CCR2 are upregulated by primary sensory neurons in lumbar ganglia by post-operative day (POD) 14. CCR2 receptor antagonist at POD 14 reverses tactile hypernociception in gp120 treated animals [98]. 

#### 3.1.3. Reactive Oxygen Species (ROS)

Oxidative stress results in activation of a number of complex and interrelated signaling events [99]. One of the pathways of oxidative stress activation is the MAPKs [100]. Increase in intracellular calcium is required for facilitation of neuronal nitric oxide synthase (nNOS) [101], leading to increased nitric oxide (NO). A host of evidence has demonstrated that free radicals have been implicated as mediators of chronic pain [102-107]. HIV-gp120 has been implicated in initiation and/or intensification of ROS and disruption of mitochondrial transmembrane potential; HIV-induced ROS regulates apoptosis signaling through TNFα and its receptors [108]. Watkins and colleagues have reported that intrathecal gp120 induces neuropathic pain and spinal release of NO as well as proinflammatory cytokines, that pretreatment with NO synthase (NOS) inhibitor abolishes gp120-induced mechanical allodynia, and that gp120-induced NO increases proinflammatory cytokines [109]. HIV gp120 induces allodynia by increasing [Ca2+]i, concomitant with activation of prostanoid EP3 and kappa-opioid receptors in the spinal cord [110]. In gp120-induced neuropathic pain model, we found that nitrated superoxide dismutase 2 (SOD2, mainly located at mitochondria) is increased in the spinal cord after peripheral gp120 application [111]. And, the activity of endogenous SOD2 is significantly downregulated by gp120 application; a new mitochondria-targeted superoxide scavenger (Mito-Tempol) significantly reverses mechanical allodynia and the decreased SOD2 activity in the model, suggesting that ROS may be a therapeutic target in the HIV-associated neuropathic pain [111] . 

#### 3.1.4. Substance P and its Receptor

The undecapeptide substance P (SP) is the prototype tachykinin and it has been identified in the central and peripheral nervous system, and in the immune system [112]. SP, as a primary sensory neurotransmitter, is found in the smaller, unmyelinated sensory ‘painful’ fibers [113]. SP is released into the dorsal horn of the spinal cord following intense peripheral stimulation, which promotes central hyperexcitability and increased sensitivity to pain [114]. Exogenous substance P, when applied to dorsal horn sensory neurons, has a slow onset and prolonged excitatory action that resembles the pattern of excitation observed after peripheral noxious stimuli [113]. The increased release of SP in the spinal cord may cause the central sensitization and hyperalgesia associated with inflammation [115, 116]. 

SP receptor (neurokinin-1 receptor, NK-1R)-expressing postsynaptic neurons in the dorsal horn of the spinal cord plays a pivotal role in the generation and maintenance of chronic neuropathic and inflammatory pain [117]. NK-1R is a potential pharmaceutical target [118]. The finding that SP is also secreted by human immune cells and participates in immunoregulation of immune cells may be of importance for the pathogenesis of AIDS [118]. SP plays a critical role in HIV gp120-induced increase in permeability of rat brain endothelium cultures, and this effect of SP on gp120-induced increase in albumin permeability is abrogated by the SP antagonists [118, 119]. Substance P enhances inflammatory cytokine (TNFα, IL-1 and IL-6) production by immune cells such as macrophages through activation of NF-κB [120]. There is a bidirectional relationship between SP and HIV infection of human immune cells [118]. In the studies of preclinical pharmacology, systemic non-peptide NK-1 receptor antagonist (SR140333B) reduces carrageenan-induced heat hyperalgesia in rats [121]. The neurokinin-1 antagonist, RP 67580, is more effective in inhibiting the behavioral response to formalin and the pain-induced activation of c-Fos [122]. But, the failure of inhibitors of NK-1R to exhibit analgesia in human, is likely due to low levels of receptor occupancy and inadequate brain penetration [123]. 

#### 3.1.5. Gamma-Aminobutyric Acid (GABA) 

Gamma-aminobutyric acid** (**GABA) is synthesized by two glutamic acid decarboxylases: GAD67 (GAD1) and GAD65 (GAD2). Between the two isoforms, GAD67 is responsible for over 90% of basal GABA synthesis and is produced at limiting levels in the brain [124]. GAD67-mediated GABA synthesis and signaling regulate inhibitory synaptic innervation [124]. The loss of GABA inhibition allows the expression of long-lasting synaptic potentiation, and by extension, the development of neuropathic pain in animals [5]. In the hippocampus, TNFα causes an endo-cytosis of GABA-A receptors, lowers surface GABA-A receptors and decreases inhibitory synaptic strength, suggesting that TNFα can regulate neuronal circuit homeostasis in a manner that may exacerbate excitotoxic damage resulting from neuronal insults [125]. We have found that peripheral gp120 application into sciatic nerve lowers the expression of GAD67 in the spinal cord and intrathecal injection of GABA-B agonist baclofen reverses mechanical allodynia induced gp120 into sciatic nerve (our unpublished data). 

#### 3.1.6. Other Pharmacological Features in the DSP Neuropathic Pain 

Nicotinic acetylcholine receptors (nAchRs) are found on peripheral monocytes, in particular the alpha seven nAchRs (α7AchRs), which when activated suppress the release of the pro-inflammatory cytokines [126, 127]. In spinal cord, α7AchRs are found on microglia. Intrathecal α7AchR agonists (GTS-21 or choline), significantly block and reverse gp120-induced mechanical allodynia, and reduce gp120-induced IL-1β protein and pro-inflammatory cytokine mRNAs within the lumbar spinal cord, supporting that α7AchRs may be a novel target for treating pain in patients with HIV [128]. HIV gp120 shed by the virus can inhibit the appropriate processing of proBDNF into mature BDNF by reducing furin levels in rat primary neurons; proBDNF, in turn, initiates neuronal damage which, in combination with other neurotoxins such as glutamate or TNFα, can lead to apoptosis and neuronal loss[129]. 

The Wnt proteins are a group of secreted lipid-modified (palmitoylation) signaling proteins. Wnt3a is up-regulated in the spinal dorsal horn of mouse pain models created by intrathecal injection of HIV-gp120 protein, suggesting that Wnt signaling pathways are involved in the nociceptive input induced by HIV-gp120 [130]. Pretreatment with intrathecal cannabilactone CB_2_R agonist AM1710 prevents bilateral mechanical hypersensitivity induced by intrathecal gp120, and reveals increased DRG IL-1β protein levels from gp120, suggesting that cannabilactone CB_2_R agonists be emerging as anti-inflammatory agents with pain therapeutic implications [131]. 

Intrathecal administration of NF-κB inhibitors, pyrrolidinedithiocarbamate (PDTC) or SN50, prior to gp120 partially attenuates gp120-induced allodynia [132]. Systemic p38 MAP kinase inhibitor CNI-1493, blocks intrathecal gp120-induced thermal hyperalgesia and mechanical allodynia [94]. Intrathecal administration of TX14(A), a 14-mer peptide derived from the C terminal region of the saposin C domain of prosaposin both prevents and alleviates intraplantar gp120-induced tactile allodynia, suggesting that the mechanism of action of TX14(A) may include modulation of spinal nociceptive processing [133]. The mechanical hypersensitivity induced by gp120 is reversed by systemic treatment with gabapentin, morphine and the cannabinoid WIN 55,212-2 [134].

Meanwhile, Acharjee and colleagues have reported the effects of a cytotoxic HIV-1 accessory protein, viral protein R (Vpr) on the peripheral nervous system, demonstrating that Vpr causes DRG neuronal damage, likely through cytosolic calcium activation and cytokine perturbation, highlighting Vpr’s contribution to HIV-associated peripheral neuropathy and ensuing neuropathic pain [135].

### 3.2. Summary

**Fig. (1):**
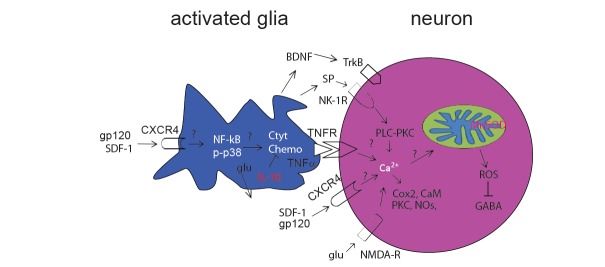
Potential HIV-related neuropathic pain pathway. Peripheral nerve inflammation after HIV infection, promotes the release of neurotransmitters and neuropeptides that stimulate glial cells in the spinal cord. The activated glia induce release of pro-inflammatory factors, such as cytokines, nerve growth factors, chemokines, glutamates, etc., which bind their receptors on the neurons to induce a massive Ca2+ influx into neurons. Ca2+ is rapidly sequestrated by mitochondria. This consequently damages MnSOD activity and increases mitochondrial ROS production, which in turn results in synaptic plasticity of the dorsal horn neurons. It is possible that ROS injures the production of GABA through GAD67 synthesis inhibition. CaM = calcium-calmodulin; Chemo=chemokines; Cyto=cytokines; glu = glutamate; NMDA-R = N-methyl-D-aspartate receptor; NO = nitric oxide.

The perivascular macrophages are the primary site of productive HIV infection. Infected macrophages or microglia release viral envelope proteins (gp120), potentially neurotoxic substances such as proinflammatory cytokines (for example, TNFα, IL-1, IL-6), chemokines, and glutamates. These neurotoxic substances stimulate astrocytosis to release similar neurotoxic factors. Many neurons express CXCR4 and CCR5, raising the possibility of direct interaction with gp120. Over activation of CXCR4 activity induces neuronal injury and allows excessive influx of Ca^2+^, then induce a host of downstream signaling pathways and second messenger systems, notably kinases (such as MAPK, protein kinase A, protein kinase C, phosphoinositide 3-kinase), which is involved in the excitability of these neurons. Based on the mechanisms, it is necessary to develop the effective and novel antagonists, for example, blockers of CXCR4, proinflammatory cytokine neutralizers and kinases inhibitors, ROS scavengers, NK-1R blockers to treat HIV neuropathic pain and neuropathy (Fig. **1**). 

## 4. ART-RELATED NEUROPATHIC PAIN

The highly active antiretroviral therapy (HAART) since 1996, dramatically has reduced the morbidity and mortality associated with HIV [136]. HAART usually contains three or more different drugs, such as two nucleoside reverse transcriptase inhibitors (NRTIs) and a protease inhibitor, two NRTIs and a non-nucleoside reverse transcriptase inhibitor or other such combinations. NRTIs decrease plasma viral load, and can result in improvements in immune function (e.g. CD4 lymphocyte count recovery) [137]. Powerful HAART has come tantalizingly close to eradicating the virus from people, driving blood level of HIV so low that standard tests cannot detect it. Therefore, HIV/AIDS have now been transformed from a rapidly progressive disease with high early mortality to a chronic disease. But no one has been cured, because the virus comes roaring back in one who stops taking the drugs. So HAART will be life-long treatment. Although the incidence of most neurological complications of HIV has fallen with HAART, rates of HIV-SN have been rising [138]. Recent estimates of HIV-SN prevalence among cohorts with access to ART range from 20% to 50% [139]. NRTI-associated painful sensory neuropathy affects quality of life in patients with HIV/AIDS. 

NRTIs (e.g., stavudine/didehydro-deoxythymidine/d4T, didanosine/2',3'-dideoxyinosine/ddI, and zalcitabine/2',3'-dideoxycytidine/ddC), are neurotoxic and cause a dose-dependent peripheral neuropathy [140]. Somehow, drug toxicity of NRTIs limits the successful treatment in many individuals (see review [137]). The ddC is used particularly for patients who were intolerant of or ineligible for AZT/ddI. The ddC is still widely used in clinics in Africa, Europe and Asia. A major dose-limiting side effect of HIV/AIDS chemo-therapies is a small-fiber painful peripheral neuropathy, mediated by mitochondrial toxicity [141]. Patients receiving HAART develop a distal symmetric small fiber retrograde (‘dying back’) axonal neuropathy with pain [38, 142-151]. However, the detailed mechanism by which these patients with HIV/AIDS experienced pain remains unknown.

### 4.1. Mechanisms of ART-Related Neuropathic Pain

#### 4.1.1. Mitochondrial Toxicity of NRTI

Mitochondrial DNA (mtDNA) is necessary for many oxidative phosphorylation complex I proteins. The mtDNA depletion causes a deficiency in complex I and an over-utilization of complex II, resulting in elevated superoxide levels [152]. Depletion in mtDNA and increased mtDNA mutations may reduce synthesis of mtDNA-encoded protein subunits required for oxidative phosphorylation [153]. Replication of mtDNA is undertaken by DNApoly-merase-γ, and this enzyme can be inhibited by NRTIs. These agents include the 2’,3’-dideoxy analogues that lack the hydroxyl radical in the 3’ position and are incorporated into DNA but prevent elongation of the DNA strand [154]. Thus, these drugs can induce mtDNA depletion and result in mitochondrial respiratory chain and oxidative phosphorylation deficits. NRTIs exert rapid toxicity by directly inhibiting mitochondrial bioenergetics function [155]. It has been estimated that significant mitochondrial DNA depletion takes place after several days to weeks of explore [156-158]. The resulting reduction in ATP and increased ROS have the potential for further mtDNA damage [159, 160]. The mitochondrial dysfunction induced by ddC alters calcium homeostasis in cultured DRG neurons [161] and in a model of ddC-associated painful peripheral neuropathy [162]. In addition, NRTIs cause direct mitochondrial toxicity through inhibition of the mitochondrial transmembrane potential differential in neurons but not in Schwann cells that are also present in the coculture [163]. Mitochondrial ultrastructural abnormalities have been noted in affected tissues, including peripheral nerves and subcutaneous tissue [151, 164, 165].

#### 4.1.2. Spinal Microgliosis

Glial cells in the CNS are essential for the maintenance of homeostasis. Activated glial cells contribute to immune deregulation and neuroinflammation, which are associated with pain and a variety of neurodegenerative disorders [166]. Spinal microgliosis, as measured by increased CD11b/c immunohistochemical staining and increased numbers of cells expressing CD11b measured by flow cytometry, is evident in the antiretroviral drug ddC or combination of perineural exposure to the HIV-gp120 protein and ddC treatment [167]. Rice and colleagues showed that ddC induced spinal microgliosis using flow cytometric quantification of OX42 (marker of microglia) [167], however, Bennett and colleagues reported that there was no microglia hypertrophy or increased Iba1 (another marker of microglia) staining in the spinal cord in the animals treated with ddC [168]. 

#### 4.1.3. Proinflammatory Cytokines, Chemokines, and ROS in NRTI-Neuropathy 

Levine and colleagues developed an animal model of NRTI-induced neuropathic pain. Systemic administration of ddC, ddI and d4T produces dose-dependent mechanical hypersensitivity and allodynia [162]. The model has been used widely to investigate the molecular mechanisms of NRTI-induced pain and the efficacy of potential therapeutic interventions. 

We have reported that systemic ddC induces mechanical allodynia and overexpression of both mRNA and proteins of glial fibrillary acidic protein (GFAP) and TNFα in the spinal dorsal horn, and that TNFα is colocalized with GFAP in the spinal dorsal horn, and with NeuN in the DRG [169]. Knockdown of TNFα with siRNA blocks the mechanical allodynia induced by ddC; intrathecal administration of recombinant TNF soluble receptor, reverses mechanical allodynia induced by ddC, suggesting that TNFα is involved in NRTI-induced neuropathic pain [169].

Systemic ddC induces the expression levels of CXCR4 mRNA in glia and neurons and SDF-1 mRNA in glia increased considerably [170]. Pain hypersensitivity produced by ddC is inhibited by treatment with the CXCR4 antagonist, AMD3100, suggesting that NRTIs produce pain hyper-sensitivity through the upregulation of CXCR4 signaling in the DRG [170]. 

Oxidative stress, which occurs in nerve tissues of patients undergoing HIV infection, is implicated in cell death of both astrocytes and neurons, and has recently been suggested to play a role in the pathogenesis of neuroAIDS [171]. Microglia produce superoxide and H_2_O_2_ upon activation in the CNS; they also produce cytokines which can enhance more production of ROS and NO [172]. Astrocytes equally produce cytokines and NO from iNOS [172]. Evidence suggests that a component of gp120 neurotoxicity may be due to increased oxidative stress [173]. Redox-regulated inflammatory pathways and synergistic proinflammatory stimulation may have significant implications in HIV-infected patients [174]. We have reported that systemic administration of ddC induces neuropathic pain and lowers the activity of endogenous SOD2 in the spinal cord dorsal horn; ROS scavengers significantly reverse mechanical allodynia in the model, suggesting that ROS systems play an important role in the neuropathic pain induced by antiretroviral therapy in patients with HIV/AIDS [175].

### 4.2. Other Preclinical Features of Pharmacology in ART-Induced Neuropathic Pain

Intradermal or spinal injection of drugs (TMB-8 and Quin-2) that buffer intracellular calcium, significantly attenuates ddC-induced mechanical hypersensitivity, suggesting that [Ca^2+^]*i* signaling plays an important role in NRTI-induced mechanical hypersensitivity [162]. Either intrathecal or peripheral TMB-8 alone significantly attenuates ddC-induced hypersensitivity by approximately 50%; furthermore, the combined administration of intradermal plus intrathecal TMB-8 almost completely eliminates ddC-induced hypersensitivity, suggesting that an abnormality in Ca^2+^ buffering at the peripheral and central terminals of primary afferent nociceptors contributes to ddC-induced painful peripheral neuropathy [162]. Peripheral administration of inhibitors of protein kinase A, protein kinase C, p42/p44-mitogen-activated protein kinase (ERK1/2) and nitric oxide synthase, which have demonstrated anti-hyperalgesic effects in other models of neurophathic pain [13, 176-179], has no effect on ddC-, ddI- and d4T-induced hypersensitivity [162]. Systemic ddC decreases conduction velocity in mechanically-evoked C-fiber activity [180]. Co-morbid conditions may contribute to this dose-limiting effect of HIV/AIDS treatment. Alcohol abuse is one of the most important co-morbid risk factors for peripheral neuropathy in patients with HIV/AIDS [141]; intradermal injection of inhibitors of the mitochondrial electron transport chain, rotenone (complex I) and oligomycin (complex V) into the hind paw, reverses the mechanical allodynia induced by ddC or by low dose of ddC plus ethanol, supporting the clinical impression that alcohol consumption enhances ART neuropathy, and providing evidence for a role of mitochondrial mechanisms underlying this interaction [141]. In addition, mitochondria exist as dynamic interconnected networks that are maintained through a balance of fusion and fission [181], which is associated with many pathological conditions, notably neurodegeneration and aging [182]. Dynamin-regulated protein 1 (Drp1) and Fis1 mediate mitochondrial fission [183, 184]. Intradermal injection of the selective Drp1 inhibitor mdivi-1 into the hind paws of rats produces a dose-dependent inhibition of ddC-induced painful hyperalgesia [185]. 

The N-type voltage-gated calcium channel (CaV2.2) is a clinically endorsed target in chronic pain treatments. Collapsin response mediator protein 2 (CRMP-2) is a novel modulator of CaV2.2 [186]. By preventing CRMP-2-mediated enhancement of CaV2.2 function, TAT-CBD3 (a peptide of CRMP-2 fused to the transduction domain of TAT protein of HIV) decreases neurotransmitter release from nociceptive DRG neurons, and reverses neuropathic hypersensitivity produced by ddC [187]. ST1-104, which disrupts the interaction between CaV2.2 and CRMP2 interaction, reduces persistent mechanical hypersensitivity induced by systemic administration of ddC [188]. Palmitoylethanolamide (PEA) is efficacious in animal models of neuropathic pain [189]. Systemic administration of L-29 (a PEA analogue) reduces mechanical hyper-sensitivity in ddC-associated hypersensitivity [190]. The mechanical hypersensitivity induced by ddC is reversed by systemic treatment with gabapentin, morphine and the cannabinoid WIN 55,212-2 [191].

Recent report shows that d4T administration to mice results in the increased neuronal activity and BDNF expression in the spinal dorsal horn and hind paws mechanical allodynia that is exacerbated by intrathecal BDNF administration. After d4T, BDNF heterozygous mice are less allodynic than wild-type littermates, which is negated by intrathecal BDNF. Blockage of BDNF-mediated signaling, significantly attenuates the development of mechanical allodynia, and decreases neuronal activity, demonstrating that BDNF in the spinal dorsal horn contributes to the development of NRTI-induced painful peripheral neuropathy and may represent a new therapeutic opportunity [192]. Rice and colleagues have reported that intravenous injections of d4T induces hind paw mechanical hypersensitivity in rats and injury to both the peripheral and central terminals of L5 dorsal root ganglion neurons, that d4T results in increased GFAP immunoreactivity, and that systemic gabapentin and cannabinoid receptor agonist WIN 55,212-2 reverse mechanical hypersensitivity in rats [193].

### 4.3. Summary

**Fig. (2):**
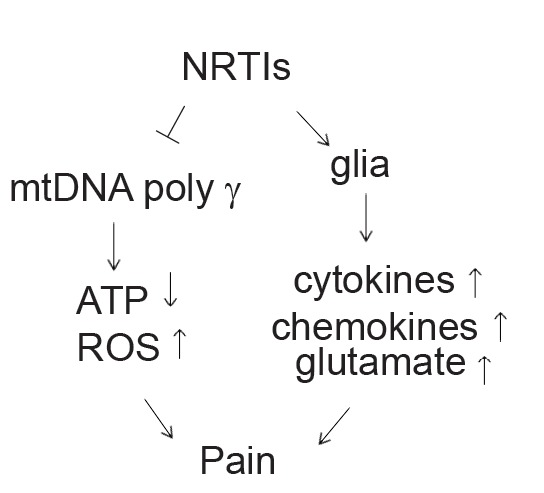
Proposed model for the role of NRTI in the HIV associated neuropathic pain. NRTIs inhibit the transcription of essential enzymes needed for ATP production by inhibiting DNA γ- polymerase. Depletion in mtDNA may reduce synthesis of mtDNAencoded protein subunits required for oxidative phosphorylation, decreasing ATP and increasing mitochondrial oxidative stress. NRTIs also activate glia to release cytokines, chemokines and BDNF to induce neuronal sensitization.

The mechanisms by which NRTIs induce neuropathic pain are not known in detail. NRTIs can alter mtDNA content by inhibiting polymerase gamma, the enzyme responsible for the replication of mtDNA [143, 151, 152, 194]. Mitochondrial DNA is necessary for many oxidative phosphorylation complex I proteins. Mitochondrial DNA depletion causes a deficiency in complex I and an over-utilization of complex II, resulting in elevated superoxide levels [152]. Studies have shown the interaction between ROS and TNFα. NRTIs also directly or indirectly induce other neurotoxic factors, such as chemokines and their receptors, MAPKs or ion channel changes, many of them are related to neuropathic pain. Therefore, understanding the molecular mechanisms is important to design new drugs to treat the NRTIs-related neuropathic pain (Fig. **2**).

## 5. MECHANISMS OF PAINFUL NEUROPATHY IN INTERACTION OF HIV INFECTION AND NRTIS 

HIV-SN in HIV/AIDS patients is associated with two types of neuropathy that are very similar clinically: distal sensory polyneuropathy, associated with advanced HIV infection per se, and antiretroviral toxic neuropathy, precipitated by the use of antiretroviral drugs in patients at varying stages of HIV infection. The onset of NRTIs-induced neuropathy is typically more acute than the onset of DSP, and pain may be more prominent [33]. In this “double-hit” condition, DRG neurons/sensory axons are damaged or sensitized by viral proteins, including gp120 and proinflammatory factors released from infiltrating HIV-infected macrophages, and are further compromised by antiretroviral drug-induced mitochondrial toxicity. Reductions in mtDNA may be caused by HIV infection alone and precede the use of NRTIs, raising the possibility that HIV directly and/or TNFα released in response to HIV infection during the immune reconstitution may injure mitochondria, potentially making them more vulnerable to the effects of NRTIs [195]. Activity in caspase signaling pathways that ultimately lead to apoptosis, plays a critical role in the generation of neuropathic pain induced by ddC and inflammatory pain induced by TNFα, before death of sensory neurons becomes apparent [196]. Therefore, closely-clinical animal model of HIV-related neuropathy should include the two factors: HIV protein toxicity and NRTIs neurotoxicity. 

Keswani and colleagues reported a rodent model of HIV-SN by oral administration of ddI to transgenic mice expressing the HIV gp120 under a GFAP promoter [197]. The neuropathy in these mice is characterized by distal degeneration of unmyelinated sensory axons, similar to the “dying back” pattern of C-fiber loss seen in patients with HIV-SN; gp120 transgenic mice treated with ddI develop mild thermal hyperalgesia [197]. 

Both perineural HIV-gp120 and systemic ddC produce many features of HIV- and anti-retroviral-related peripheral neuropathy, and that combination of peripheral gp120 and ddC induces an enhanced mechanical allodynia [191]. The combination of gp120 and ddC induces inflammatory response in sciatic nerve and DRG, which is in line with clinic studies indicating that patients with HIV show a certain degree of inflammation involvement in the DRG [38]; combination of gp120 and ddC induces loss of epidermal nerve fibers, which is seen in HIV/AIDS patients [39]. Moreover, a spinal gliosis is apparent at times of peak behavioral sensitivity that is exacerbated in gp120+ddC as compared to either treatment alone. Finally, the hyper-sensitivity to mechanical stimuli is sensitive to systemic treatment with gabapentin, morphine and the cannabinoid WIN 55,212-2. These data therefore merit further investigation for the elucidation of underlying mechanisms and may prove useful for preclinical assessment of drugs for the treatment of HIV-related peripheral neuropathic pain [191]. 

MCP1 (known as CCL2) and stromal derived factor-1 (SDF1/CXCL12) and their respective receptors, CCR2 and CXCR4, have been implicated in HIV-related neuropathic pain mechanisms including NRTI treatment in rodents [170]. White and colleagues reported that gp120 sciatic nerve injury in combination with ddC at day 14 after gp120 produces pronounced bilateral tactile hypernociception [98]. More importantly, functional MCP1/CCR2 and SDF1/CXCR4 signaling is present in sensory neurons. CXCR4 antagonist AMD3100 effectively reverses the hypernociceptive behavior associated with the gp120+ddC, indicating that the functional upregulation of CCR2 and CXCR4 signaling systems following a combination of gp120 plus NRTI is likely to be of central importance [98]. 

Our recent studies and others have demonstrated that either gp120 application into sciatic nerve or systemic ddC activates spinal glia using immunohistochemistry and Western blots [86, 134, 169]. Blackbeard and colleagues have reported that gp120+ddC induces spinal microgliosis using flow cytometric quantification or immunohistochemistry [167, 191]. The degree of mechanical hypersensitivity is found to be in line with spinal cord microgliosis in gp120+ddC as determined by CD11b/c cytometry [167]. BDNF in the spinal dorsal horn contributes to the development of NRTI-induced painful peripheral neuropathy [192]. Rice and colleagues, have reported that the truncated isoform of TrkB, a receptor for BDNF, is the most upregulated gene in the gp120+ ddC model using conventional analysis and Gene Set Enrichment Analysis [198].

Alterations in calcium activity may be related to an additive/synergistic effect of a combination of gp120 and ddC exposure. Intradermal or spinal injection of intracellular calcium modulators significantly attenuates ddC-induced mechanical hypersensitivity, suggesting the intracellular calcium dysfunction induced by ddC is likely involved in ddC-associated painfully peripheral neuropathy [162]. HIV gp120 increases [Ca^2+^]_i_ in populations of the cultured DRG neurons through CXCR4 [56]. The mitochondrial dysfunction induced by ddC alters calcium homeostasis in cultured DRG neurons [161] and in ddC-induced neuropathic pain [162]. HIV gp120 may induce axonal degeneration directly through mitochondrial caspase pathway and indirectly through neuronal apoptosis mediated by the activation of Schwann cells [199]. Such an additive/synergistic effect of the treatments with gp120+ddC in behavioral indices of pain may be related with intracellular calcium mitochondrial dysfunction [98, 191]. 

In summary, HIV-associated sensory polyneuropathies induced by HIV infection and antiretroviral therapy share clinic symptoms. Animal models of NRTI-induced neuropathy have yielded similar molecular mechanisms compared to that induced by HIV gp120 protein in the neuropathic pain state. Models of combined NRTI and HIV gp120 protein-induced neuropathy have provided similar molecular mechanisms, mainly including the proinflammatory cytokines, chemokine, oxide stress, MAPK and calcium channel changes, many of them are interacted and twisted together and reciprocal relationship in the molecular mechanisms.

## CONCLUSION

HIV associated neuropathic pain is a symptom across the entire process of the disease, and remains a common and debilitating symptom frequently reported in clinics. This review sums up the current advance of molecular mechanisms, suggesting that glia activity induced neurochemical factors, such as, proinflammatory cytokines, chemokines, and neurotrophic factor, ROS, calcium channels, and MAP kinases, play important roles in the development of the neuropathic pain. As the mechanisms underlying HIV-related neuropathic pain are better understood, focusing on the molecular targets (e.g., receptor blockers of cytokines and chemokines, scavengers of ROS, etc.) will be valuable for developing novel pharmaceutical approaches to the treatment of HIV-neuropathic pain. Indeed, the precise mechanisms are not fully known, and the preclinical models need to be improved. Therefore, the more detailed molecular mechanisms have yet to be investigated, such as the relationship between neurotoxic factors, regions of molecular targets in the nervous system, and interaction of glia and neurons, which will have the potential to open new pharmaceutical approaches.
